# Burned area mapping using satellite data – a tool for monitoring effects on environment

**DOI:** 10.1093/eurpub/ckac130.072

**Published:** 2022-10-25

**Authors:** ML Cara, A Becheru, L Nicola, C Cara, IM Prejbeanu

**Affiliations:** 1 Public Health, University of Medicine and Pharmacy, Craiova, Romania; 2 Computers & Information Technolgy, University of Craiova, Craiova, Romania; 3 Software Solutions for Applied Science, CS GROUP - ROMANIA, Craiova, Romania; 4 Department of Hygiene, University of Medicine and Pharmacy, Craiova, Romania

## Abstract

**Background:**

Significant crop residue burning not only negatively affects local communities but is becoming an important public health issue for global climate change mitigation efforts. This practice is linked with air quality impairment, water contamination, soil degradation, fauna destruction. Chronic exposure to a high level of air pollution may cause permanent health injuries such as the development of lung diseases. The intentional burning of crop residues is a well-known practice across Romania even it is restricted by law.

**Methods:**

We propose a generic software model capable of detecting burned areas based on a time series of multi-spectral optical images together with a multi-step algorithm that uses a pre-trained Gaussian Naive-Bayes classifier to map burned crop fields, named BCMA (Burned Crop Mapping Algorithm), using Copernicus Sentinel-2 acquisitions. BCMA can be trained and extended to recognize other user-defined burned areas and it can be used to produce burned crop fields maps at a global scale in near-real-time at high resolution. We focused on two restrained geographical areas where burned areas were signalized in local press as massive burning vegetation events.

**Results:**

We provide burned area maps generated with an implementation of BCMA over two sites in Romania: Ostroveni - Dolj county and Domogled - Mehedinti county, based on acquisitions from July 2020.

**Conclusions:**

This model provides a fast and reliable tool for detection of burned areas regardless of the landscape and vegetation that could help stakeholders to react and make a proper intervention. Our study highlights the meaningful implications of using this tool to track crop fields burning and to organize large scale awareness campaigns around sustainable crop residues management with positive impact on environment, human health, and agriculture. Further government agencies’ positions regarding using satellite monitoring of burning vegetables might be crucial.

**Key messages:**

- Satellite imagery is a valuable source of information for environment monitoring.
- More strategies are need to bring health and environment sustainability to the center of the climate change and to deliver information to the policy makers.

